# The Effect of Remaining Coronal Tissue Height on the Fracture Strength of Over-Flared Endodontically Treated Central Incisors Restored Using a Multipost Approach

**DOI:** 10.1155/2023/3681815

**Published:** 2023-08-29

**Authors:** Mahsa Farshbaf-antik, Mahdi Abed-Kahnamouei, Parnian Alizadeh-Oskoee, Narmin Mohammadi, Siavash Savadi-Oskoee

**Affiliations:** ^1^Department of Operative Dentistry, Faculty of Dentistry, Tabriz University of Medical Sciences, Tabriz, Iran; ^2^Dental and Periodontal Research Center, Tabriz University of Medical Sciences, Tabriz, Iran

## Abstract

**Objectives:**

This study aimed to examine the effect of remaining coronal tissue height on the fracture strength of over-flared endodontically treated central incisors restored with multiple prefabricated fiberglass posts using the multipost approach.

**Materials and Methods:**

A total of 40 human central maxillary incisors were examined in this study. The samples were assigned to five groups (*n* = 8) based on the height of the remaining coronal tissue: with no remaining coronal tissue, 1-mm coronal tissue height (CTH1), 2-mm coronal tissue height, 3-mm coronal tissue height (CTH3), and one intact tooth (IT) group. Following endodontic treatment of an over-flared canal, the postspace depth was 10 mm, and the residual dentin thickness was 1 mm. Two prefabricated fiberglass posts were cemented into the root canal, adopting a multipost approach. The static load was applied at 0.5 mm/min and 135° concerning the tooth's longitudinal axis until a fracture occurred. One-way analysis of variance and the post hoc Tukey's test were performed to analyze the data at a significance level of *p* < 0.05.

**Results:**

The maximum fracture strength was recorded for IT (control group), while the minimum fracture strength was found for teeth with a coronal tissue height of 1 mm. The differences between IT group and other groups (*p* < 0.05), as well as the differences between the group with CTH3 and groups without coronal tissue and CTH1, were significant.

**Conclusion:**

In sum, an increase in the height of the remaining coronal tissue (≥3 mm) significantly increased the fracture strength of over-flared endodontically treated central incisors after restoration with prefabricated fiberglass posts by adopting a multipost approach.

## 1. Introduction

Clinicians have always faced serious challenges when deciding on restoration of endodontically treated teeth with weak coronal structures [1, 2]. Posts are employed to provide the coronal restoration mechanical strength and retention when the tooth's remaining coronal tissue is not sufficient [3, 4]. Various post and core systems are adopted to restore endodontically treated central incisors. Furthermore, endodontically treated central incisors are often restored using metal posts and cores [4]. Recently, prefabricated posts have become more popular in terms of accessible clinical applications [5, 6]. Fiber-reinforced resin composites reduce the incidence of nonrestorable root fractures due to their esthetic properties, ease of use, biomechanical properties similar to dentin, and ability to homogeneously distribute the exerted stresses. However, the low elastic modulus of fiberglass posts compared to the metal types leads to a significant accumulation of stresses on the cervical one-third and increases fracture incidence in the restoration core region [4–7]. Debilitation of the tooth root structure following caries, trauma, pulp pathology, and some iatrogenic factors during canal preparation increases the risk of root fracture and diminishes the long-term prognosis of the treatment [8]. Several restoration techniques have been developed for dealing with central incisors with over-flared canals undergoing endodontic treatment, including reinforcement of root canal with composite resin, application of bonded fibers, direct or indirect fabrication of anatomical posts, and adoption of accessory fiber posts using multipost approach. However, the application of accessory posts or multipost strategies employing fiber posts has attracted more attention [9, 10]. Although no definite conclusion has been reached concerning the most effective technique due to the inconsistencies in results from different studies, the multipost technique is a time- and labor-saving technique that provides more favorable fracture patterns than single postplacement conditions [8].

The strength and survival of teeth restored with post and core depend on the type of postmaterial and forces exerted on the teeth, thickness, height of the root wall, and presence of remaining coronal tissue (ferrule) [11]. According to an accepted convention, the ferrule is the most important mechanical factor contributing to the strength of endodontically treated teeth [2]. However, the clinical outcomes are heavily affected by the extent of the remaining coronal tissue. Due to the sloped direction of imposed pressures as well as the deleterious tensile/compressive and shear stresses on the maxillary incisors, this is stressed when choosing the most appropriate restoration approach [2–12]. Considering the importance of a successful restoration of over-flared endodontically treated central incisors with high degradation of coronal tissue, the role of remaining tissue height in fracture resistance, and treatment success [12]—especially when there is a time gap between crown restoration and crown placement, this study aimed to examine the effect of remaining coronal tissue height on the fracture strength of over-flared endodontically treated central incisors under restoration conditions by adopting the multipost approach.

## 2. Materials and Methods

Table 1 lists the properties of the materials used in the present study. The study protocol was approved by the ethical committee. Forty intact human maxillary incisors, extracted due to periodontal diseases, with no caries, crack, or fracture (magnification 4x), as well as with the same root length and size and similar internal anatomy based on radiographic images (buccolingually and mesiodistally) were collected from donors. A total of 40 teeth with similar buccolingual root dimensions of 8 ± 0.5 mm and length of 14 ± 0.6 mm distance from the buccal cementoenamel junction (CEJ) to apex were selected after performing measurement using a digital caliper (Mitutoyo Corporation, Kanagawa, Japan) and periapical films. The total sample size was determined using PASS (sample size software) and data from prior research with a similar experimental design [13]. The suggested sample size was at least eight specimens for each group. The samples were kept in a phosphate-buffered solution until the initiation of the study [14]. Finally, they were assigned to five groups of eight specimens at the time of investigation, including four groups assigned based on the height of the remaining coronal tissue with no remaining coronal tissue (NCT), 1-mm coronal tissue height (CTH1), 2-mm coronal tissue height (CTH2), and 3-mm coronal tissue height (CTH3), as well as one group assigned based on intact tooth (IT). The first four groups were sectioned based on the residual coronal tissue's height after measuring the area above buccal CEJ using a digital caliper (Mitutoyo Corporation, Kanagawa, Japan) and a slow-rate diamond saw (Diaswiss, Geneva, Switzerland) [15].

The root canals were prepared by using step-back technique and stainless steel K-files (Dentsply Maillefer Instruments SA, Ballaigues, Switzerland) and #4, #3, #2, #1 Gates-Glidden drills (Mani, Tokyo, Japan) as well as by obturation with Gutta Percha (Meta Biomed Gutta Percha, South Korea) and AH26 sealer (Dentsply, Konstanz, Germany) adopting lateral compaction technique. Then, root canal orifices of the roots were sealed by conventional glass ionomer (Ketac-Bond; 3M ESPE, Sumaré, SP, Brazil) and kept at 37°C with relative humidity 100% for 48 hr [15]. A 10-mm postspace was prepared for each sample using #1, #2, #3, #4 Gates-Glidden drills (Mani, Tokyo, Japan) and #1 Exacto standardized bur (Angelus, Brazil). Next, the dentin wall of the root was weakened using a taper diamond bur (#850 023) (Diaswiss, Geneva, Switzerland) until the remaining dentin was 1-mm thick. The residual dentin thickness was measured using a digital caliper with 0.01 accuracy.

In order to standardize the dentin wall thickness, reference points were marked on the cervical area using a periodontal probe [15]. After over-flaring the root canal, radiographic examinations were repeated from the buccolingual and mesiodistal directions to evaluate the uniform thickness of the remaining root canal dentin. The roots were exposed to humid gas during dentin wall thinning. A layer of molten wax with a thickness of 0.2–0.3 mm up to 3-mm CEJ was placed onto the root surface in order to simulate the periodontal ligament conditions. Then, the teeth were placed in a cylinder filled with polystyrene resin (Cristal, Piracicaba, SP, Brazil) as mold. After resin polymerization, the teeth were removed from the cylinder, and hot water was used to remove the wax around the root surface. Then, polyether molding material (Impregum, 3M USA) was poured into the resin cylinder using a molding syringe, and the tooth was placed again inside the cylinder socket. Polyether was placed inside the wax-filled space to simulate a periodontal ligament with a thickness of 0.2–0.3 [13–15]. After 1 min of silanization (Silano, Angelus, Brazil) and applying 96% ethanol, two prefabricated fiberglass posts, including primary Number #1 fiber with a coronal diameter of 1/4 mm (Exacto, Angelus, Brazil) and one accessory fiber (Reforpin, Angelus, Brazil) with a coronal diameter of 1.3 mm, were simultaneously cemented into the root canal using dual-cure resin cement (duo-LINK Universal; Bisco Inc., Schaumburg, IL, USA) according to the manufacturer's instruction. A light cure device (Bluephase 20i, Ivoclar Vivadent, Schaan, Liechtenstein) with an intensity of 1,200 mW/cm^2^ was used for 40 s according to the manufacturer's instructions [16]. A two-step etch-and-rinse adhesive (Single Bond 2, 3M Oral Care) was used to bond the coronal area. Standardized cores of 5.0 mm in height were built using a transparent polyethylene core buildup matrix (CoreForms, KaVo Kerr, Brea, CA, USA) and a light-cure resin composite (Gradia Direct GC, Tokyo, Japan) in increments. Each 2.0-mm increment was light-cured for 20 s [16].

All samples were kept in an environment with a humidity of 100% for 24 hr. After completion of 1,500 heat cycles between 5 and 60°C for two 20-s periods, each sample was positioned on a fixed metal device and subjected to static load at 0.5 mm/min with an angle of 135° to the tooth longitudinal axis in the universal testing machine (Hounsfield Test Equipment, H5K-S Model; Surrey, England) until a fracture occurred. The round-tip cylindrical-shaped device (2.0 mm in diameter) attached to the load cell was used as a palatal load (2 mm from the palatal incisal edge) [13]. Data distribution was determined by performing the Shapiro–Wilk normality test. The fracture strength data were analyzed using one-way analysis of variance (ANOVA) and post hoc Tukey's test at a significance level of *p* < 0.05. Statistical analysis was performed using the SPSS 21 software (SPSS Inc., Illinois, USA).

## 3. Results

The Shapiro–Wilk normality test results were indicative of normal distribution in this in vitro study. The mean and standard deviation of the study groups' fracture strength values are shown in Table 2. One-way ANOVA findings showed significant differences among the five study groups in terms of fracture strength (*p* < 0.05); IT (control group) and CTH1 group had the maximum and minimum fracture strengths, respectively. The results from post hoc Tukey's pairwise comparison test (Table 3) revealed significant differences between CTH3 and NCT (*p* < 0.05), CTH3 and CTH1 (*p* < 0.05), as well as IT (control group) and all other study groups including NCT (*p* < 0.05), 1CTH (*p* < 0.05), 2CTH (*p* < 0.05), and 3CTH (*p* < 0.05). There were no significant differences among other group pairs.

## 4. Discussion

Dentists face great challenges when deciding on restoration of the endodontically treated central incisors with extensive coronal degradation and over-flared conditions [2, 3]. Debilitation of the biomechanical properties of teeth following extensive coronal degradation (more than 50%) requires using a post to enhance the coronal restoration retention and resistance [17, 18]. The role of fiber-reinforced resin composites in enhancing the fracture strength of coronal restorations has been confirmed due to the similar biomechanical characteristics of dentin, the ability to evenly distribute the applied stresses [19], and acceptable long-term clinical performance [5–20]. Under the over-flaring conditions in root canal therapy, in addition to the unfavorable stress absorption by the weak dentin wall of the root [21], increasing the volume of luting cement consumed to fill the space between the post and canal increases.

The low strength of luting cement, features, and entrapment of bubbles at high volumes of luting material [22], as well as polymerization shrinkage of different types of resin—under C factor conditions above 200 inside the root, in particular [23]—all contribute to compromising the fracture strength and clinical lifespan of the tooth. A multipost technique using fiber post in over-flared endodontically treated central incisors reduces the resin cement volume and, as a result, its polymerization shrinkage as well as increases the post surface area and the interface between the post and core. It also decreases the stress resulting from polymerization shrinkage and pull-out risk in terms of increased retention without needing further drilling of the root dentin [24]. These results have been associated with enhanced fracture strength of weakened roots [25–27].

The results from studies indicated that restorations placed on several reinforcing posts at a certain distance from the tooth's longitudinal axis acted like a reinforced structure capable of withstanding high intrusive/extrusive and bending forces [24]. In other words, placing the fiber posts in the same direction and at a certain distance from the longitudinal axis of the endodontically treated teeth provides appropriate conditions contributing to stress creation, transfer, and distribution similar to that seen in natural tooth. Therefore, the risk of root fracture in the apical area is minimized [8]. Regardless of the fiberglass post types, their elastic modulus, which is comparable to resin and dentin composite systems, makes their combination with polymerized resin cement a mono-block structure in the root. This combination increases the strength of the endodontically treated tooth to that of an IT by reducing stress accumulation and better stress distribution [8–25]. According to our study results, the highest fracture strength was observed in the IT despite the increase in fracture strength of over-flared endodontically treated teeth restored adopting the multipost approach technique.

Another critical point in endodontically treated central incisors is the extent of remaining coronal tissue, which may influence the treatment prognosis [12]. Our study, therefore, investigated the influence of the residual coronal tissue height on the fracture strength of over-flared endodontically treated central incisors restored with multiple prefabricated fiberglass posts using a multipost approach. The findings of the static test (fracture strength) showed a significant difference between the CTH3 group and CTH1 and between the CTH3 and NCT in terms of in fracture strength. In other words, the fracture strength in the samples with coronal tissue height of 3 mm was significantly higher than that in the other two groups. This result was in line with findings from other studies demonstrating that the endodontically treated maxillary teeth with remaining coronal tissue of at least 2 mm were restored conservatively by resin composites as core buildup and had a higher fracture resistance and a greater chance of restorable fracture [28–31].

Santos-Filho et al. [12] observed that endodontically treated anterior teeth restored (cast post and core) with a ferrule height of 2 mm had higher fracture resistance compared to teeth with no ferrule. This increase in the wall height up to 3–4 mm was associated with a considerable increase in fracture resistance. Corrêa et al. [4] examined the effect of thickness and height of the remaining coronal tissue and found that regardless of restoration strategies (fiber post against cast post and core), the samples without remaining coronal structure with less than 1 mm of residual thickness had a lower survival rate than the teeth with remaining coronal structure [4]. Our study results showed that an increase in ferrule height was necessary for teeth with compromised roots and 1-mm thickness to provide the desired fracture strength.

The remaining coronal height (ferrule) is responsible for enhancing resistance and changing the bending behavior of restored teeth [1]. Under inclined forces exerted at 45°, the anterior teeth undergo tensile, compressive, and shear stresses. These inclined forces consist of a horizontal component with induction of static load and a vertical component with bending effect induction. All these factors lead to the formation of tensile, compressive, and shear stresses along the buccal and lingual surfaces and center of repaired teeth, which ultimately deteriorates the set of teeth and restoration [4]. The presence of a cervical ferrule, which shifts the location of the fulcrum and lessens the impact of bending and the length of the lever arm, prevents fracture and even fracture progression [4–28]. In this regard, a finite-element study showed that increasing the ferrule height by reducing the axial arm of rotational forces and, as a result, the tensile stresses decreased the risk of fracture of fiber-reinforced composite (FRC) post/core restorations [12–16]. On the other hand, the remaining coronal tissue increased the strength and retention of FRC posts with its protective effect against functional forces, the wedging effect of conical posts, and side forces created during posting [2–30]. According to 3-year clinical follow-ups of Mancebo et al. [11–32], only 6.67% of the endodontically treated incisors with coronal tissue height of 2 mm and resorted with the post were degraded. However, this value increased to 26.2% in teeth with no coronal tissue. A meta-analysis study investigated the effect of the loss of remaining coronal tissue on the fracture rate of FRC post/core restorations, whose results showed that the absence of coronal tissue increases the risk of restoration fracture [25]. A notable point in our study results was the low fracture strength values in the group without ferrule and restored with a multipost approach, which was in line with the results of Magne et al. [31]. They explored the effect of ferrule against the application of fiber posts in endodontically treated central incisors and found that the presence of a ferrule reduced the severity of tooth fracture. However, using fiber posts failed to enhance the load-bearing ability and longevity of the restoration tooth complex as well as to counteract the adverse effects of the ferrule's absence. All given studies confirmed the effect of the remaining coronal height (ferrule) on the fracture strength of endodontically treated teeth. Similarly, in the present study, the maximum fracture strength was recorded for the IT group. A significant difference was reported elsewhere between IT and teeth with severe coronal degradation regarding fracture resistance [33, 34].

In sum, measuring fracture resistance by adopting destructive methods similar to those employed in the present study did not always follow in vivo conditions. In such tests, static compressive force is used; in other words, the speed, value, and direction of force are constant and are continuously applied until a fracture occurs. However, the forces in the mouth are dynamic, meaning that their magnitude, speed, and direction may change over time. Furthermore, repeated stresses in the mouth may cause cusp fatigue failure. Therefore, it was recommended that our study results, as well as those from related studies, should be closely evaluated. It was also suggested that longitudinal research in clinical settings should be carried out before generalization of the findings.

## 5. Conclusions

Despite the limitations of our study, it was concluded that adopting the multipost approach by using prefabricated fiberglass posts and increasing the height of the remaining coronal tissue (≥3 mm) increased the fracture strength of crownless over-flared endodontically treated incisors.

## Figures and Tables

**Table 1:** Properties of the materials used in the present study.

Material	Composition	Manufacturer
Exacto	87% glass fiber 13% epoxy resin	Angelus, Londria, PR, Brazil
Reforpin Universal	80% glass fiber 20% epoxy resin	Angelus, Londria, PR, Brazil
Duo-Link	BIS-GMA, TEGDMA, UDMA, 62% Ultrafine glass filler	Bisco, Inc, Schaumburg, IL, USA
Single Bond 2	10% Silica filler size 5 nm	3M ESPE Dental Products, St Paul, MN, USA
Silano	X-R-Si (OR)3n X: Organofunctional group. R: Methylene group. OR: Hydrolyzable group Si: Silicon	Angelus, Londria, PR, Brazil
AH 26	Powder: Bismuth oxide, Methenamine Liquid: Bisphenol epoxy resin	Dentsply, Konstanz, Germany
Gradia anterior direct	Urethane di-methacrylate (UDMA) 25%–50% di-methacrylate 5%–10% tri-methacrylate 5%–10%	GC America Inc., US

**Table 2:** Mean and standard deviation of fracture strength values (*N*) in the study groups.

	No.	Mean	Standard deviation	*p*-Value
With no coronal tissue	8	497.66	194.39	<0.001
Coronal tissue with a 1 mm height	8	465.57	127.80	
Coronal tissue with a 2 mm height	8	662.65	107.99	
Coronal tissue with a 3 mm height	8	849.47	141.07	
Human intact tooth	8	1218.60	369.37	

**Table 3:** Tukey test for paired comparison of the groups in terms of fracture strength.

(*I*)	(*J*)	Mean difference (*I* − *J*)	*p*-Value	Confidence interval 95%	
				Lower limit	Upper limit
NCT	CTH 1	32.09	>0.001	−288.13	352.32
NCT	CTH2	−164.99	>0.001	−474.36	144.38
NCT	CTH3	−351.81	≤0.001	−672.03	−31.58
NCT	IT	−720.94	≤0.001	−1030.31	−411.57
CTH1	CTH2	−197.08	>0.001	−517.30	123.15
CTH1	CTH3	−383.90	≤0.001	−714.63	−53.17
CTH1	IT	−753.03	≤0.001	−1073.25	−432.80
CTH2	CTH3	−186.82	>0.001	−507.05	133.40
CTH2	IT	−555.95	≤0.001	−865.32	−246.58
CTH3	IT	−369.13	≤0.001	−689.35	−48.90

## Data Availability

The data used to support the findings of this study are included within the article.
